# Syncope in the setting of trifascicular block and retrograde concealed conduction: A case report

**DOI:** 10.3892/mi.2023.124

**Published:** 2023-11-23

**Authors:** Dimitrios Sfairopoulos, Christos S. Konstantinou, Panagiotis Korantzopoulos

**Affiliations:** First Department of Cardiology, University of Ioannina Faculty of Medicine, 45110 Ioannina, Greece

**Keywords:** trifascicular block, left posterior hemiblock, premature ventricular contractions, concealed conduction, syncope

## Abstract

In clinical practice, the accurate diagnosis of the causes of syncope is often challenging and demanding. Moreover, certain rare electrocardiographic phenomena may complicate the diagnostic workup, leading to imprecise diagnoses. The present study briefly describes the case of an 82-year-old male patient with ischemic cardiomyopathy who suffered syncopal episodes in the setting of trifascicular block. The 12-lead electrocardiogram revealed premature ventricular contractions and non-conducted P waves due to the phenomenon of retrograde concealed conduction. Following the exclusion of myocardial ischemia, an electrophysiological study yielded abnormal results and a biventricular pacemaker was implanted. Although retrograde concealed conduction is considered a benign phenomenon caused by the transient modification of antegrade atrioventricular conduction characteristics, further meticulous investigation is required in patients with concomitant baseline conduction abnormalities and/or structural heart disease.

## Introduction

Syncope is a common health concern with significant clinical consequences and diverse underlying etiologies. In clinical practice, the accurate diagnosis of the causes of syncope is often challenging and demanding (1,2). Moreover, some rare electrocardiographic phenomena may complicate the diagnostic workup, leading to imprecise diagnoses. Undoubtedly, a specific diagnosis is a prerequisite for an effective management plan (1,2).

The present study briefly describes the case of an 82-year-old male patient with ischemic cardiomyopathy who suffered syncopal episodes in the setting of trifascicular block.

## Case report

**Figure 1:**
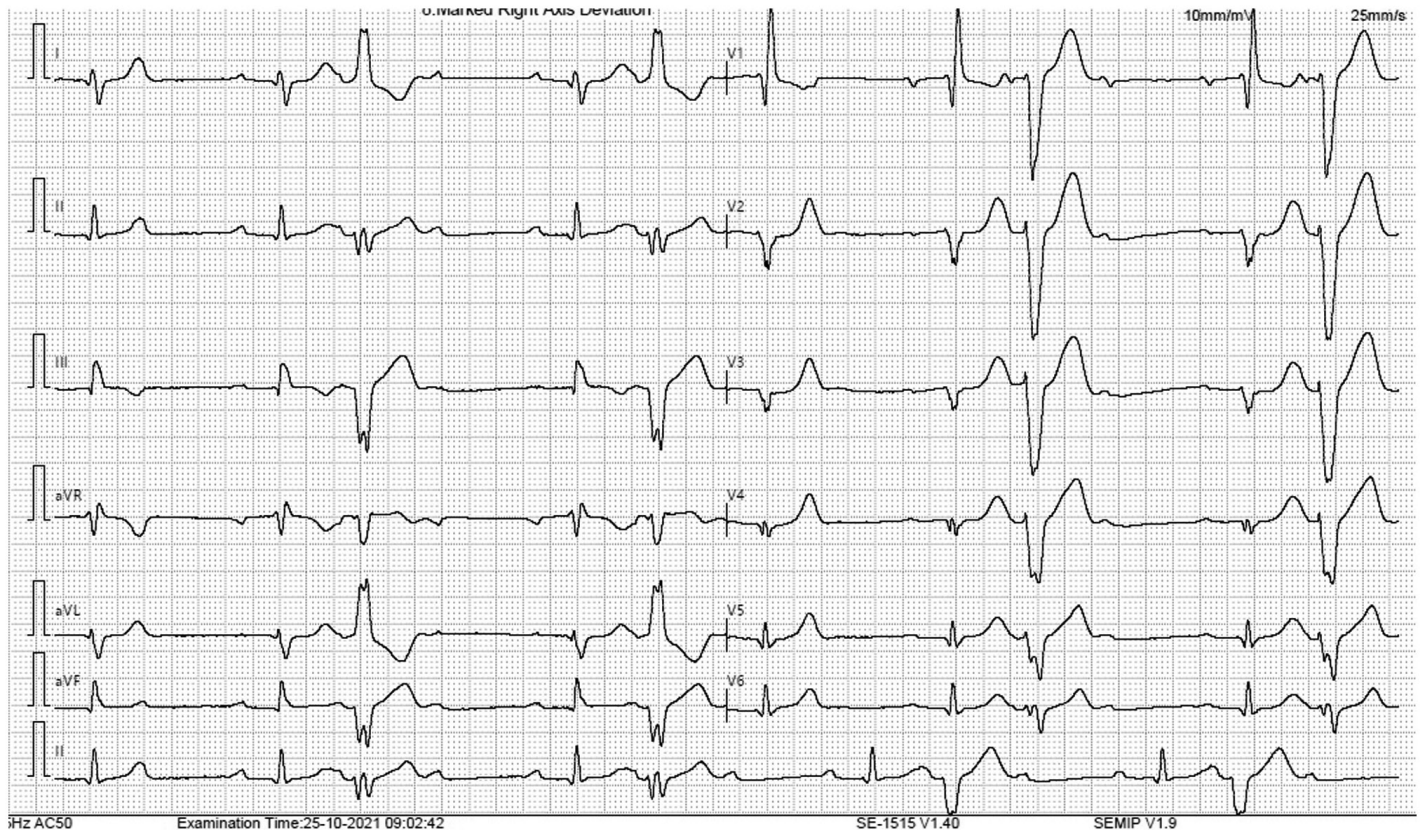
The 12-lead electrocardiogram of the patient upon admission.

An 82-year-old male patient with a history of anterior myocardial infarction and hypertension suffered two syncopal episodes in the sitting position without prodromal symptoms during the previous few weeks. His medications included bisoprolol, valsartan, aspirin, and atorvastatin. The patient was referred to The First Department of Cardiology, University Hospital of Ioannina, Ioannina, Greece for further evaluation by his general physician. His baseline 12-lead electrocardiogram (ECG) revealed sinus rhythm, first-degree atrioventricular block, right bundle branch block, left posterior hemiblock (LPH), Q wave in leads V1-V5 (consistent with the old myocardial infarction), and ventricular bigeminy (Fig. 1). Of note, two different morphologies of premature ventricular contractions (PVCs) were evident, raising the suspicion of an ischemic substrate. Interestingly, after each PVC, a non-conducted sinus beat was evident (Fig. 1). This form of atrioventricular block is not generally considered pathological, since it is explained by the phenomenon of retrograde concealed conduction. An echocardiogram revealed a left ventricular ejection fraction (LVEF) of 40%, anterior wall akinesia, and mild mitral regurgitation. A myocardial perfusion single photon emission computed tomography did not demonstrate reversible myocardial ischemia. Moreover, electrocardiographic monitoring for 24 h did not reveal any bradycardia events or episodes of second- or third-degree atrioventricular block. However, similar to the baseline ECG, several episodes of ventricular bigeminy were observed, while the burden of PVCs was 15% of the total beats.

Although an immediate implantation of a permanent pacemaker would be a sensible approach based on the clinical history and the electrocardiographic findings of trifascicular block and LPH (which is not benign), an electrophysiological study was first performed, given the presence of ischemic cardiomyopathy with moderately depressed LVEF in order to exclude a predisposition to malignant ventricular arrhythmias. Of note, the programmed ventricular stimulation failed to induce ventricular tachycardia. However, the HV interval was 90 msec, and the Wenckebach point was at a cycle length of 650 msec (92 bpm). Based on these findings, as well as on the depressed LVEF and the anticipated high burden of ventricular pacing, a biventricular pacemaker was implanted. Furthermore, medical treatment for heart failure was optimized, and the dose of beta-blocker was up-titrated, leading to amelioration of the burden of PVCs. At 12 months after the implantation, the patient was clinically stable with a slightly improved LVEF (45%), an effective biventricular pacing level of 97%, and without any atrial or ventricular arrhythmias recorded by the device diagnostics. No further syncopal episodes were noted, while the daily level of PVCs during the last device interrogation was <2%.

## Discussion

Bearing in mind that the patient in the present study had trifascicular block with LPH, the latter being associated with structural heart disease and/or significant pathology in the conduction system (3), it is possible that the dropped sinus beats may have been falsely attributed to atrioventricular block. However, these non-conducted sinus beats occurred only after the PVCs. This rare phenomenon is due to the incomplete retrograde penetration of the atrioventricular node by the PVCs, causing a transient modification of its antegrade conduction characteristics (4,5). Of note, the retrograde electrical stimulation of the atrioventricular node is not directly apparent on the ECG, as it is ‘concealed’. However, it affects the subsequent conduction patterns, causing increased refractoriness of the atrioventricular node, manifested either as a prolonged PR interval in the subsequent conducted sinus beat or as a block of the next sinus beat, as observed in the case described herein (2,3).

Indeed, the consequences of retrograde concealed conduction of a PVC may vary depending on whether there is concomitant antegrade intranodal excitation, as well as on the degree of retrograde penetration (4,6). Therefore, the variable response of the atrioventricular conduction/atrioventricular refractoriness following retrograde concealed conduction may vary from a simple transient prolongation of the PR interval (variable PR intervals may be observed) to a completely blocked atrial beat (5,6). Notably, both phenomena can be observed during a continuous electrocardiographic recording of a particular patient (5). Another phenomenon that may ensue due to retrograde concealed conduction of a PVC is a temporary nodal escape rhythm with atrioventricular dissociation (6).

Retrograde concealed conduction per se is not an indication for permanent pacemaker implantation. Atrioventricular block due to retrograde concealed conduction is very transient and usually does not cause symptoms, while beta-blocker therapy can be continued and even up-titrated for the suppression of the PVCs (5). In the case presented herein, this phenomenon was evident in the setting of trifascicular block in a patient with ischemic cardiomyopathy and moderately reduced LVEF who suffered syncopal episodes. Therefore, after exclusion of myocardial ischemia, further evaluation with an electrophysiological study was performed, revealing severe conduction abnormalities in the atrioventricular node and His-Purkinje system, while malignant ventricular arrhythmias were not induced. Of note, in patients with impaired atrioventricular conduction and a diseased His-Purkinje system, retrograde concealed conduction may aggravate these abnormalities (7).

In conclusion, even though retrograde concealed conduction is considered a benign phenomenon, further meticulous investigation is required in patients with concomitant baseline conduction abnormalities and/or structural heart disease.

## Data Availability

The datasets used and/or analyzed during the current study are available from the corresponding author on reasonable request.
